# Correction: Increased survival and cell cycle progression pathways are required for EWS/FLI1-induced malignant transformation

**DOI:** 10.1038/s41419-018-0773-9

**Published:** 2018-07-23

**Authors:** Tahereh Javaheri, Zahra Kazemi, Jan Pencik, Ha T. T. Pham, Maximilian Kauer, Rahil Noorizadeh, Barbara Sax, Harini Nivarthi, Michaela Schlederer, Barbara Maurer, Maximillian Hofbauer, Dave N. T. Aryee, Marc Wiedner, Eleni M. Tomazou, Malcolm Logan, Christine Hartmann, Jan P. Tuckermann, Lukas Kenner, Mario Mikula, Helmut Dolznig, Aykut Üren, Günther H. Richter, Florian Grebien, Heinrich Kovar, Richard Moriggl

**Affiliations:** 1 Ludwig Boltzmann Institute for Cancer Research, Vienna, Austria; 2 Institute of Animal Breeding and Genetics, University of Veterinary Medicine, Vienna, Austria; 3 Medical University of Vienna, Vienna, Austria; 4 Center of Physiology and Pharmacology, Vienna, Austria; 5 Clinical Institute of Pathology, Vienna, Austria; 6 Children’s Cancer Research Institute, St. Anna Kinderkrebsforschung, Vienna, Austria; 7 CeMM Research Center for Molecular Medicine of the Austrian Academy of Sciences, Vienna, Austria; 8 Department of Pediatrics, Medical University of Vienna, Vienna, Austria; 9 Randall Division of Cell and Molecular Biophysics, King’s College London, London, UK; 10 Department of Bone and Skeletal Research, Institute of Experimental Musculoskeletal Medicine, University Hospital Münster, Münster, Germany; 11 Institute of General Zoology and Endocrinology, University of Ulm, Ulm, Germany; 12 University of Veterinary Medicine, Vienna, Austria; 13 Institute of Medical Genetics, Vienna, Austria; 14 Georgetown University Medical Center, Lombardi Comprehensive Cancer Center, Washington, USA; 15 Children’s Cancer Research Centre and Department of Pediatrics, Klinikum rechts der Isar, Technische Universität München, Munich, Germany

**Correction to:** *Cell Death and Disease* (2016) **7**, e2419; 10.1038/cddis.2016.268, Published online 13 October 2016.

Since the publication of the article, the authors became aware that Fig. 6a, d contained errors in the bands and loading controls. The newly compiled Fig. 6a, d is given below.

**Fig. 6:**
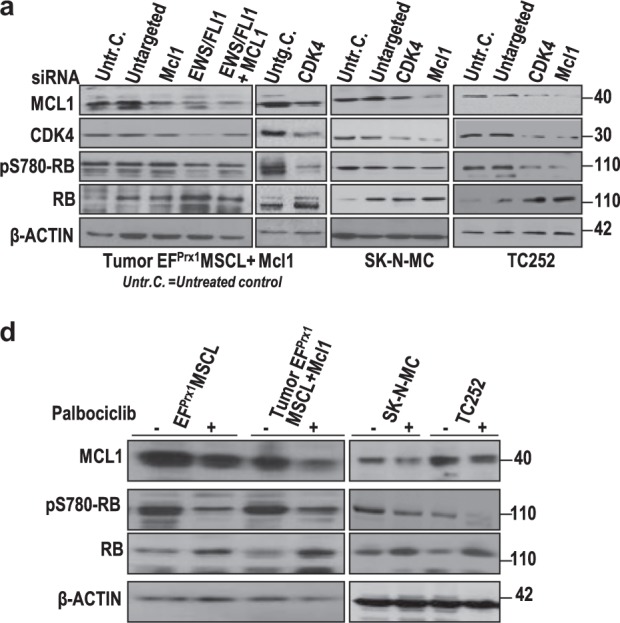
Knockdown of Mcl1 or Cdk4-induced cell cycle arrest and inhibited ES cell proliferation. **a** Mcl1 and Cdk4 siRNA analysis and expression check in murine and human ES cell lines resulted in upregulation of total RB and lower amount of pS780-RB. **b** Colony-forming assay in Matrigel displayed reduced clonogenic potency of EF-dependent cell lines after knockdown of Mcl1 or Cdk4 by siRNA treatment. **c** Cell cycle analysis of Mcl1 or Cdk4 siRNA treatment displayed enhanced G1 arrest. **d** In vitro growth inhibition of mouse and human EF-dependent cell lines by Palbociclib as well as the combinatorial treatment of Palbociclib and Obatoclax in triplicate. Cells were treated with inhibitors at the indicated concentration for 24 h. **e** CDK4/6 inhibition by Palbociclib displayed similar results as seen in the CDK4 siRNA assay

The authors apologise for any inconvenience caused.

